# Retraction Note: Hsa-miR-623 suppresses tumor progression in human lung adenocarcinoma

**DOI:** 10.1038/s41419-019-1300-3

**Published:** 2019-01-25

**Authors:** Shuang Wei, Zun-yi Zhang, Sheng-ling Fu, Jun-gang Xie, Xian-sheng Liu, Yong-jian Xu, Jian-ping Zhao, Wei-ning Xiong

**Affiliations:** 1 Department of Respiratory and Critical Care Medicine, Key Laboratory of Pulmonary Diseases of Health Ministry, Key Cite of National Clinical Research Center for Respiratory Disease, Tongji Hospital, Tongji Medical College Huazhong University of Science and Technology, 1095 Jie Fang Avenue, 430030 Wuhan, China; 2 Department of Surgery, Tongji Hospital, Tongji Medical College Huazhong University of Science and Technology, 1095 Jie Fang Da Dao, 430030 Wuhan, China

**Retraction Note to:** **Cell Death and Disease** (2016) 7: e2388

10.1038/cddis.2016.260

published online 29 Sept 2016

The authors have retracted the article [Hsa-miR-623 suppresses tumor progression in human lung adenocarcinoma, Cell Death & Disease volume 7, page e2388 (2016), 10.1038/cddis.2016.260] because it has recently come to their attention that the A549 cells used in this research were contaminated with Hela cells, which may have altered the outcome of their experiment. The conclusions of this article are therefore unreliable. All authors agree to this retraction.

